# Homeless Girls

**Published:** 1873-01

**Authors:** 


### HOMELESS GIRLS.

I KNOW a little girl who, with a worthy mother, walked the streets of New York all night because they had no place to sleep, having been turned out of their lodgings by the improvidence of an intemperate husband.

I know a clergyman, without a stain on his name, who slept in a cart all night in New York city, because he had not the means to pay for a lodging; and is now actively engaged in good doing.

Many little boys sleep all night in store boxes and house stoops and under wagons and butcher's carts; but in mid-winter the houseless poor must suffer sadly. If any reader of these lines has a heart to help a little, he can send it to No. 27 St. Mark's place, New York, where one night's shelter and a plain breakfast are offered to any houseless girl under eighteen years of age. A printed ticket of admission is obtained at the Children's Aid Society, No. 193 Fourth street, which begins at 696 Broadway; a ticket is not given until means have been used to ascertain that the applicant is worthy of assistance. If the reader is living in the country it should be remembered that a large number of the applicants are from the country. Surely such a charity ought not to be stinted in its operations for want of funds; your daughter, reader, your cousin, some relative might need such aid one of these days, yes, may have received such aid already.

There is a similar place for men in Pearl street, provided they are destitute and are honestly endeavoring to find work. The accomodations are offered but for a single night to the same person, as it is not intended to encourage idleness and unthrift.

